# Editorial: Understanding and Bridging the Gap Between Neuromorphic Computing and Machine Learning

**DOI:** 10.3389/fncom.2021.665662

**Published:** 2021-03-17

**Authors:** Lei Deng, Huajin Tang, Kaushik Roy

**Affiliations:** ^1^Department of Precision Instrument, Center for Brain Inspired Computing Research (CBICR), Tsinghua University, Beijing, China; ^2^College of Computer Science and Technology, Zhejiang University, Hangzhou, China; ^3^Zhejiang Lab, Hangzhou, China; ^4^Department of Electrical and Computer Engineering, Purdue University, West Lafayette, IN, United States

**Keywords:** neuromorphic computing, spiking neural networks, machine learning, artificial neural networks, cross paradigm

## Introduction

On the road toward artificial general intelligence (AGI), two solution paths have been explored: neuroscience-driven neuromorphic computing such as spiking neural networks (SNNs) and computer-science-driven machine learning such as artificial neural networks (ANNs). Owing to availability of data, high-performance processors, effective learning algorithms, and easy-to-use programming tools, ANNs have achieved tremendous breakthroughs in many intelligent applications. Recently, SNNs also attracted a lot of attention due to its biological plausibility and the possibility of achieving energy-efficiency (Roy et al., 2019). However, they suffer from ongoing debates and skepticisms due to worse accuracy compared to “standard” ANNs. The performance gap comes from a variety of factors, including learning techniques, benchmarks, programming tools and execution hardware, all of which in SNNs are not as developed as those in the ANN domain.

To this end, we propose a Research Topic, named “Understanding and Bridging the Gap between Neuromorphic Computing and Machine Learning,” in Frontiers in Neuroscience and Frontiers in Computational Neuroscience to collect recent researches on neuromorphic computing and machine learning to help understand and bridge the aforementioned gap. We received 18 submissions in total and accepted 14 of them in the end. The scope of these accepted papers covers learning algorithms, applications, and efficient hardware.

## Learning Algorithms

How to train SNN models is the key to improve its functionality, thus bridging the gap between ANN models. Unlike the ANN domain that has grown rapidly via sophisticated backpropagation-based learning algorithms, the SNN domain is still short of effective learning algorithms due to the complicated spatiotemporal dynamics and non-differentiable spike activities. Currently, there are overall two categories of learning algorithms for SNNs: unsupervised synaptic plasticity with biological plausibility [e.g., spike timing dependent plasticity, STDP (Diehl and Cook, 2015)] and supervised backpropagation with gradient descent [e.g., indirect learning by acquiring gradients from the ANN counterpart (Diehl et al., 2015; Sengupta et al., 2019), direct learning by acquiring gradients from the SNN itself (Lee et al., 2016; Wu et al., 2018; Gu et al., 2019; Zheng et al., 2021), or the combination of both (Rathi et al., 2020)]. The latter can usually achieve higher accuracy and has advanced the model scale to handle ImageNet-level tasks. In the future, we look forward to seeing more studies on SNN learning to close the gap.

Next, we briefly summarize the recent progress of neural network (especially SNN) learning presented in our accepted papers. Inspired by the curiosity-based learning mechanism of the brain, Shi et al. propose curiosity-based SNN (CBSNN) models. In the first training epoch, the novelty estimations of all samples are obtained through bio-plausible synaptic plasticity; next, the samples whose novelty estimations exceed the threshold are repeatedly learned and the novelty estimations are updated in the next five epochs; then, all samples are learned with one more epoch. The last two steps are periodically taken until convergence. CBSNNs show better accuracy and higher efficiency in processing several small-scale datasets than conventional voltage-driven plasticity-centric SNNs. Daram et al. propose ModNet, an efficient dynamic learning system inspired from the neuromodulatory mechanism in the insect brain. An inbuilt modulatory unit regulates learning based on the context and internal state of the system. The network with modulatory trace achieves 98.8% ± 1.16 on average over the omniglot dataset for five-way one-shot image classification task while using 20x fewer trainable parameters compared to state-of-the-art models. Kaiser, Mostafa et al. introduce deep continuous local learning (DECOLLE), an SNN model equipped with local error functions for online learning. The synaptic plasticity rules are derived from user-defined cost functions and neural dynamics by leveraging existing autodifferentiation methods of machine learning frameworks. The model demonstrates state-of-the-art performance on N-MNIST and DvsGesture datasets. Fang et al. propose a bio-plausible noise structure to optimize the performance of SNNs trained by gradient descent. Through deducing the strict saddle condition for synaptic plasticity, they demonstrate that the noise helps the optimization escape from saddle points on high dimensional domains. The accuracy improvement can reach at least 10% on MNIST and CIFAR10 datasets. Panda et al. modify the SNN configuration with backward residual connections, stochastic softmax, and hybrid artificial-and-spiking neuronal activations. In this way, the previous learning methods are improved with comparable accuracy but large efficiency gains over the ANN counterparts.

## Applications

Unlike the artificial neuron in ANNs, each spiking neuron in SNNs has intrinsic temporal dynamics, which is appropriate for processing sequence information. In this Research Topic, we accepted two papers that discuss SNN applications. Wu et al. explore the first work that uses SNNs for large-vocabulary continuous automatic speech recognition (LVCSR) tasks. Their SNNs demonstrate competitive accuracies on par with their ANN counterparts while consuming only 10 algorithmic timesteps and 0.68× total synaptic operations. They integrate the models into the PyTorch-Kaldi Speech Recognition Toolkit for rapid development. Kugele et al. apply SNNs for processing spatiotemporal event streams (e.g., N-MNIST, CIFAR10-DVS, N-CARS, and DvsGesture datasets). They improve the ANN-to-SNN conversion learning method by introducing connection delays during the pre-training of ANNs to match the propagation delays in converted SNNs. In this way, the resulting SNNs can handle the above tasks accurately and efficiently.

In addition, besides energy-efficiency (Merolla et al., 2014), recent studies further find that the event-driven computing paradigm of SNNs endows them high robustness (He et al., 2020; Liang et al., 2020) and superior capability in learning sparse features (He et al., 2020). We believe it is very important to mine the true advantages of SNNs to determine their true value in practical applications.

## Efficient Hardware

Performing neural networks on general-purpose processors is inefficient, which stimulates the development of various domain-specific hardware platforms, including those for ANNs [e.g., DaDianNao (Chen et al., 2014), TPU (Jouppi et al., 2017), Eyeriss (Chen et al., 2017), Thinker (Yin et al., 2017), etc.), for SNNs (e.g., SpiNNaker (Furber et al., 2014), TrueNorth (Merolla et al., 2014), Loihi (Davies et al., 2018), DYNAPs (Moradi et al., 2017)], and for cross-paradigm modeling [e.g., Tianjic (Pei et al., 2019; Deng et al., 2020)]. In this Research Topic, we accepted seven papers for neural network hardware: three for ANNs, two for SNNs, and two for cross-paradigm.

Sim and Lee propose SC-CNN, the bitstream-based convolutional neural network (CNN) inspired by stochastic computing (SC) that uses bitstreams to represent numbers, to improve machine learning hardware. Benefitting from the CNN substrate, SC-CNN can achieve high accuracy; further benefitting from SC, SC-CNN is highly efficient, scalable, and fault-tolerant. Different from the common digital machine learning accelerators, Kaiser, Faria et al. present a clockless autonomous probabilistic circuit, wherein synaptic weights are read out in the form of analog voltages, for fast and efficient learning with no use of digital computing. They demonstrate a circuit built with existing technology to emulate the Boltzmann machine learning algorithm. Muller et al. introduce bias matching, a top-down neural network design approach, to match the inductive biases required in a machine learning system to the hardware constraints of its implementation.

To alleviate the high cost training of SNNs using backpropagation, Lee et al. propose a spike-train level direct feedback alignment (ST-DFA) algorithm. Compared to the state-of-the-art backpropagation learning algorithm, they demonstrate excellent performance vs. overhead tradeoffs on FPGA for speech and image classification applications. Dutta et al. propose an all ferroelectric field-effect transistors (FeFET)-based SNN hardware that allows low-power spike-based information processing and co-localized memory and computing. They implement a surrogate gradient supervised learning algorithm on their efficient SNN platform, which further accounts for the impacts of device variation and limited bit precision of on-chip synaptic weights on the classification accuracy.

Parsa et al. build a hierarchical pseudo agent-based multi-objective Bayesian hyperparameter optimization framework for both software and hardware. They can not only maximize the performance of the network, but also minimize the energy and area overheads of the corresponding neuromorphic hardware. They validate the proposed framework using both ANN and SNN models, which involves both deep learning accelerators [e.g., PUMA (Ankit et al., 2019)] and neuromorphic hardware [e.g., DANNA2 (Mitchell et al., 2018) and mrDANNA (Chakma et al., 2017)]. Instead of implementing ANNs and SNNs separately, integration of them has become a promising direction to achieve further breakthroughs toward AGI via complementary advantages (Pei et al., 2019). Therefore, the efficient hardware that can support individual modeling of ANNs and SNNs as well as their hybrid modeling is very important. This has been achieved by the cross-paradigm Tianjic platform (Deng et al., 2020), based on which Wang et al. further present an end-to-end mapping framework for implementing various hybrid neural networks. By constructing hardware configuration schemes for four typical signal conversions and establishing a global timing adjustment mechanism among different heterogeneous modules, they implement hybrid models with low execution latency and low power consumption.

## Conclusion

Machine learning and neuromorphic computing are two modeling paradigms on the road toward AGI. ANNs have achieved tremendous breakthroughs in many intelligent applications benefitting from big data, high-performance processors, effective learning algorithms, and easy-to-use programming tools; in contrast, SNNs are still in its infant stage and there is a dire need for more neuromorphic benchmarks. Through cross-discipline research, we expect to understand and bridge the gap between neuromorphic computing and machine learning. This Research Topic is just a small step in this direction, and we look forward to more innovations that can achieve brain-like intelligence.

## Author Contributions

All authors listed have made a substantial, direct and intellectual contribution to the work, and approved it for publication.

## Conflict of Interest

The authors declare that the research was conducted in the absence of any commercial or financial relationships that could be construed as a potential conflict of interest.
